# Bidirectional associations between word memory and one-legged balance performance in mid and later life

**DOI:** 10.1016/j.exger.2020.111176

**Published:** 2021-02

**Authors:** Joanna M. Blodgett, Rachel Cooper, Daniel H.J. Davis, Diana Kuh, Rebecca Hardy

**Affiliations:** a MRC Unit for Lifelong Health and Ageing at UCL, London, UK; b Musculoskeletal Science and Sports Medicine Research Centre, Department of Sport and Exercise Sciences, Manchester Metropolitan University, Manchester, UK; c CLOSER, Social Research Institute, UCL, London, UK

**Keywords:** Cognitive ageing, Physical performance, Balance, Epidemiology, Life course, Birth cohort

## Abstract

**Background:**

Age-related changes in cognitive and balance capabilities are well-established, as is their correlation with one another. Given limited evidence regarding the directionality of associations, we aimed to explore the direction and potential explanations of associations between word memory and one-legged balance performance in mid-later life.

**Methods:**

A total of 3062 participants in the Medical Research Council National Survey of Health and Development, a British birth cohort study, were included. One-legged balance times (eyes closed) were measured at ages 53, 60–64 and 69 years. Word memory was assessed at ages 43, 53, 60–64 and 69 with three 15-item word-recall trials. Autoregressive cross-lagged and dual change score models assessed bidirectional associations between word memory and balance. Random-effects models quantified the extent to which these associations were explained by adjustment for anthropometric, socioeconomic, behavioural and health status indicators.

**Results:**

Autoregressive cross-lagged and dual change score models suggested a unidirectional association between word memory and subsequent balance performance. In a sex-adjusted random-effects model, 1 standard deviation increase in word memory was associated with 9% (7,12%) higher balance performance at age 53. This association decreased with age (−0.4% /year (−0.6,-0.1%). Education partially attenuated the association, although it remained in the fully-adjusted model (3% (0.1,6%)).

**Conclusions:**

There was consistent evidence that word memory is associated with subsequent balance performance but no evidence of the reverse association. Cognitive processing plays an important role in the balance process, with educational attainment providing some contribution. These findings have important implications for understanding cognitive-motor associations and for interventions aimed at improving cognitive and physical capability in the ageing population.

## Highlights

• Associations between memory and balance from age 43 to 69 were tested.
• There were positive associations between memory and subsequent balance performance.
• Education partially explained these associations, other factors did not.
• There was no evidence of association between balance and subsequent memory.

## 1 Introduction

As both cognitive and balance abilities decline with age, associations between measures are frequently found. Whether these associations are simply an artefact of their correlations with age or whether changes in cognitive capability precipitate changes in balance ability or vice versa has not been established. Understanding the temporality and direction of the relationship between these measures has important implications for interventions aimed at improving cognitive and balance outcomes. Previous research on this topic has been limited by a focus on aggregate physical or cognitive measures (Clouston et al., 2013), the application of traditional regression models that only allow the investigation of unidirectional associations (Chen et al., 2016; Tolea et al., 2015), and little consideration of underlying pathways of associations (Finkel et al., 2016). Evidence on bidirectional associations is sparse, despite plausible reasons to expect that associations may act in one or both directions.

Firstly, there may be shared underlying factors driving decline in cognitive and balance abilities (Christensen et al., 2001) such that any observed associations could simply be a consequence of simultaneous declines with age. Equally, functional decline in areas of the brain responsible for integrating sensory and motor information could impact an individual's balance ability. This may include fronto-parietal areas, the right cerebellum, and basal ganglia structures (Emch et al., 2019). The cerebellum is the coordination centre of the brain and regulates posture, movement and balance (Morton and Bastian, 2004), while the basal ganglia are primarily responsible for regulating motor control, but also play a key role in learning and executive function (Bostan and Strick, 2010). Similar to the cerebellum, the basal ganglia form iterative synaptic loops with the cerebral cortex, which are involved in movement and cognitive function (Leisman et al., 2014; Middleton and Middleton and Strick, 2000).

There may also be indirect pathways that act in both directions. Those with higher cognitive ability are more likely to have more advantaged socioeconomic position, healthier behaviours and positive health outcomes, each of which are associated with better balance (Birnie et al., 2011; Blodgett et al., 2020b; Richards and Richards and Sacker, 2011; Singh-Manoux et al., 2005). Equally, poor balance could lead to activity restriction, decreased social activity and lower physical health, which could subsequently impact cognitive ability (McDermott and McDermott and Ebmeier, 2009; Singh-Manoux et al., 2003; Zunzunegui et al., 2002).

We used longitudinal data from the MRC National Survey of Health and Development to test i) bidirectional associations between balance performance and word memory between ages 43 and 69 years and ii) potential explanatory socioeconomic, behavioural or health pathways. We hypothesised that there would be bidirectional associations between word memory and balance and that associations between memory and subsequent balance would be independent of other pathways, while associations between balance and subsequent memory would be largely explained by socioeconomic, behavioural and health pathways.

## 2 Materials and methods

This study followed the Strengthening the Reporting of Observational Studies in Epidemiology (STROBE) guidelines (von Elm et al., 2007).

### 2.1 Study sample

The MRC National Survey of Health and Development (NSHD) is a nationally representative, age-homogenous sample of 5362 individuals born within one week in March 1946 in England, Scotland and Wales. Study members have been followed from birth, for up to 24 waves, with the most recent data collection at age 69 years. Study member retention has been high and reasons for non-participation have been previously described (Kuh et al., 2016; Stafford et al., 2013). Briefly, of 5362 study members at birth, 3062 (57.1%) had at least one measure of balance and one measure of word memory at any age and were included in analyses. By age 69, the remaining study members had either died (*n* = 726;13.5%), emigrated (*n* = 543;10.1%), permanently or temporarily refused to participate (*n* = 1012;18.9%) or had missing balance (*n* = 11;0.2%) or word memory data (*n* = 8;0.1%) at all ages. Relevant ethical approval and written informed consent was provided at all waves. Approval for the most recent visit at age 69 was given by Queen Square Research Ethics Committee (13/ LO/1073) and Scotland A Research Ethics Committee (14/SS/1009).

### 2.2 Measurement of word memory and balance

Word memory, historically called *verbal memory* in NSHD (Richards and Sacker, 2003), was assessed at ages 43, 53, 60–64 and 69 years by research nurses using a 15-item word-learning task devised by the NSHD team. Using a rotating card index, each word was presented by the nurse to the participant for 2 seconds. Once all fifteen words had been presented, participants had 1 minute to write down all of the words that they could remember. The score (range: 0–45) represents the number of words correctly recalled over three identical trials. To minimise any practice effects, two word lists were rotated such that a different list was used at the subsequent follow-up. Each of the fifteen words were unrelated to one another; examples include imagine, wheat and hotel. This test was chosen a priori as the measure of cognition because repeat data were available over four waves in NSHD and previous evidence demonstrated robust associations of this specific cognitive measure with balance performance (Blodgett et al., 2020a; Kuh et al., 2009; Saverino et al., 2016).

*Balance performance* was assessed by research nurses at ages 53, 60–64 and 69 using a one-legged balance test. Individuals were instructed to cross their arms, stand on their preferred foot and raise their other leg a few inches off the ground. Participants were given the opportunity to practice once. The nurse stopped timing a) when the raised leg touched the floor as the participant lost their balance or b) after a maximum of 30 s. One trial was completed with eyes open followed by a trial with eyes closed. Due to a ceiling effect for balance times with eyes open in NSHD as in other studies of middle-aged adults (Morioka et al., 2012; Springer et al., 2007), eyes closed scores were used for analysis. The one-legged balance test with eyes closed is a reliable measure of static balance, with high inter-rater (ICC: 0.98–1.00) and test-retest reliability (ICC: 0.72–0.74) (Franchignoni et al., 1998; Kammerlind et al., 2005; Michikawa et al., 2009; Springer et al., 2007) and is associated with a range of adverse outcomes (Choy et al., 2007; Cooper et al., 2010; Cooper et al., 2014; El-Sobkey, 2011). Individuals who were unable to complete the test due to health reasons were allocated a balance time of 0 s (Blodgett et al., 2020a).

### 2.3 Measurement of covariates

Time-varying covariates were measured at ages 53, 60–64 and 69 years. This includes ascertainment of height (cm) and weight (kg) using standard protocols by research nurses (Braddon et al., 1986), which were used to calculate BMI (kg/m^2^), as well as self-reported measures of leisure-time physical activity (never, 1–4 times/month, 5+ times/month), smoking status (never, ex, current smoker), history of diabetes (yes, no), history of cardiovascular events (yes, no), respiratory symptoms (yes, no)(Cooper et al., 2014) and symptoms of depression and anxiety (28-item General Health Questionnaire; range: 0–84)(Goldberg and Hillier, 1979). Occupational class was self-reported at age 53 using the Registrar General's Social Classification (I-Professional/ II-Intermediate, III-Skilled non-manual or manual, IV-Partly skilled/ V-Unskilled manual) (Galobardes et al., 2006). The highest level of educational attainment by age 26 was self-reported as degree or higher, advanced secondary qualifications (generally attained at 18 years), ordinary secondary qualifications (generally attained at 16 years), below ordinary secondary qualifications, or none. Binary indicators of death and non-death attrition were included to minimise bias resulting from poorer performance in those lost to follow-up (Botoseneanu et al., 2013). Death was dichotomised as alive at age 69 or died between ages 53 and 69. Attrition was dichotomised as participating in the study at age 69 or attrition not due to death between ages 53 and 69.

### 2.4 Statistical analyses

Sample characteristics by sex are described for median balance times, mean word memory scores and all covariates at each age, with differences assessed by Kruskal-Wallis, *t*-tests or chi-square tests respectively. Three distinct modelling techniques were employed: autoregressive cross-lagged models, bivariate dual change score models and random-effects models. Analyses were conducted in Stata 14.0 and Mplus v6.1 (maximum likelihood estimator with robust standard errors (Grimm et al., 2017)).

**Fig. 1:**
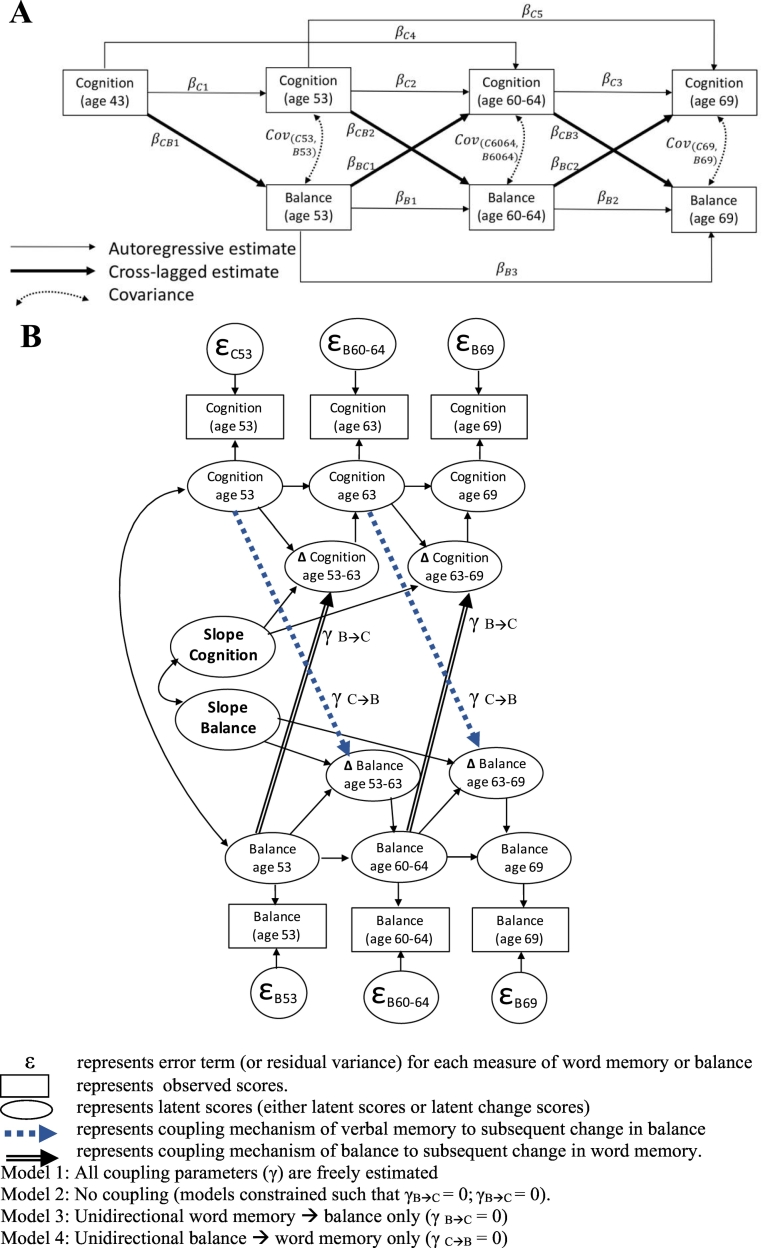
Analytical model demonstrating A. all pathways of the autoregressive cross-lagged model and B. all model terms and coupling mechanisms of the bivariate dual change model.

*Autoregressive cross-lagged models* assessed directional associations between word memory and balance over time. Auto-regressive components describe the stability of each construct over time, cross-lagged components describe reciprocal associations over time and covariance estimates allow for the expected correlation of variance between cognition and balance at each age (Fig. 1A) (Selig and Selig and Little, 2012). Satorra-Bentler Scaled chi-square difference tests are used to compare pathways (e.g. reciprocal cross-lagged pathways at the same age, unidirectional pathways at multiple ages, pathways between males and females) (Satorra and Bentler, 2001). Standardised estimates (per 1SD) are presented. Full information maximum likelihood, assumes that data are missing at random, and utilises all available variables to estimate the model regardless of an individual's missing data (Collins et al., 2001).

*Bivariate dual change score models* combine aspects of growth modelling and autoregressive cross-lagged models to evaluate the extent to which balance performance or word memory can predict change in the other (Grimm et al., 2017). Latent scores, latent change scores (e.g. ∆ between two ages) and growth curves (based on the change scores) are estimated for each of balance performance and word memory, while the addition of coupling parameters (γ_B➔C_ and γ_C➔B_) allows change in one construct to depend on previous scores in the other (e.g. change in balance from age 53 to 60–64, word memory at age 53) (Fig. 1B). First, a full model with all coupling parameters is freely estimated. Next, the coupling parameters are constrained to be zero, denoting no association between constructs. The third model tests the unidirectional association of word memory to balance and the fourth the unidirectional association in the opposite direction. By constraining coupling parameters, hypotheses about the direction of association are tested by examining the fit of four different models; the model with the best fit can provide hypotheses about the direction of association. The following indices are used to assess model fit: Comparative Fit Index (CFI), Tucker-Lewis Index (TLI), Root Mean Square Error of Approximation (RMSEA), Standardised Root Mean Square Residual (SRMR), Akaike Information Criterion (AIC) and Bayesian Information Criterion (BIC).

*Random-effects models* use random intercepts and slopes to partition the total variation into that attributable to individual factors and to changes over time within the same individual (Lininger et al., 2015). Dependent on the results of the bidirectional models, lagged random-effects models assessed the association between the independent variable at *time x* (e.g. age 53) with the dependent variable at *time x* *+* *1* (e.g. age 60–64), with age centred at age 53 (e.g. intercept). Initial models assessed for non-linearity and age or sex interactions of the independent variable and any covariates; interactions or non-linear terms were included if *p* < 0.05. An initial sex-adjusted model adjusted for death and attrition between ages 53 and 69, followed by anthropometric measures. Using this base model, each stage of adjustment additionally considered health status indicators, health behaviours, adult social class and education. A final fully-adjusted model is also presented.

## 3 Results

### 3.1 Sample characteristics

**Fig. A.1:**
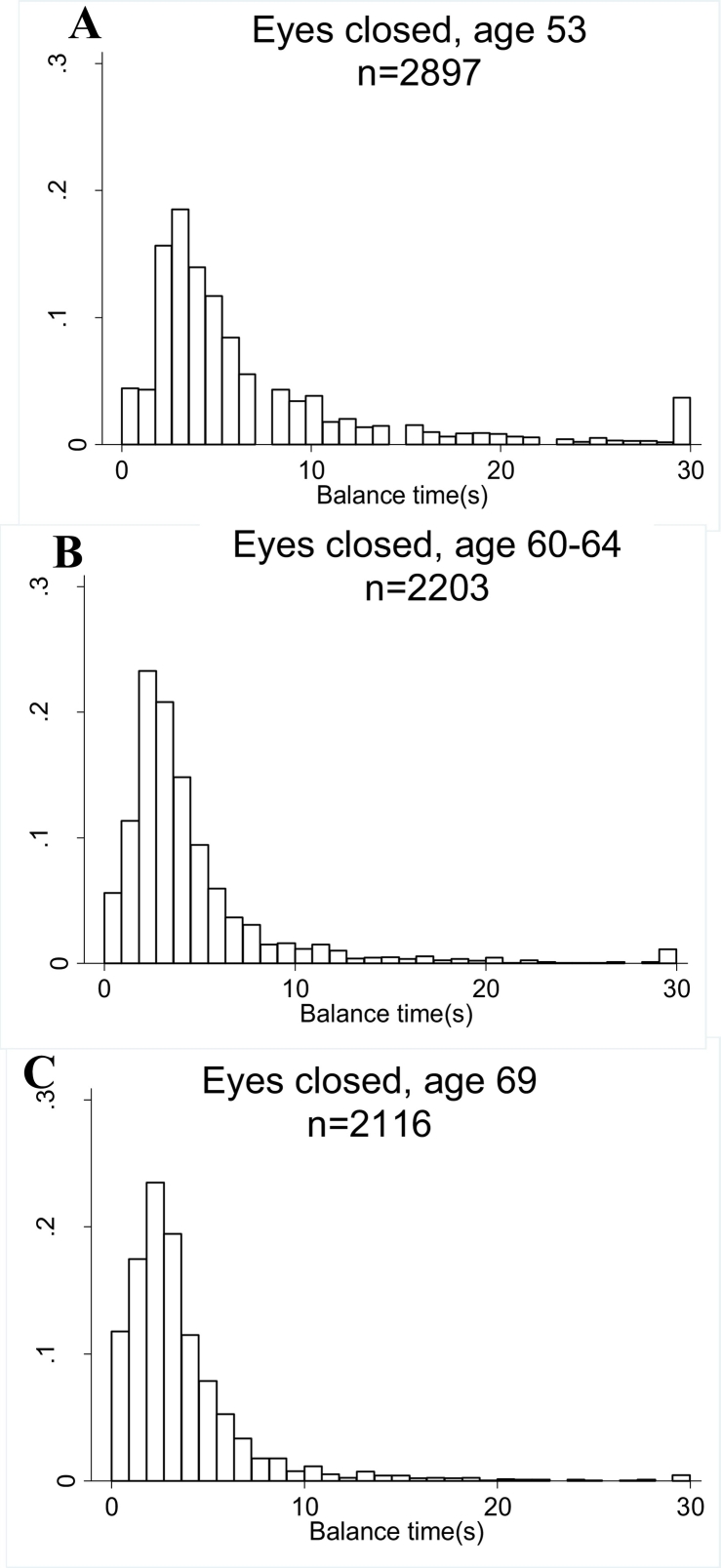
Distribution of balance times among maximal sample at ages 53, 60–64 and 69 years.

The distribution of balance times was right skewed as the majority of individuals could not maintain the position with eyes closed for more than 10 s (Fig. A.1). At all ages, males had higher median balance times than females, while females had higher mean word memory scores. Males were also more likely than females to be taller, be a current or ex-smoker, have a history of CVD events, and have higher occupational class and educational attainment. Conversely, females were more likely to have knee pain and more symptoms of depression/anxiety (Table 1).

**Table 1:** Characteristics of maximal analytical sample (up to *n* = 3062), MRC National Survey of Health and Development.

	Males (*n* = 1515)	Females (*n* = 1547)	Tests of sex differences (*p*-value)
Balance time (S), median (Q1, Q3), n			a
Age 53	5 (3,10), *n* = 1389	4 (3, 7), *n* = 1464	<0.001
Age 60–64	3.57 (2.35, 5.53), *n* = 1047	3.16 (2.16, 4.72), *n* = 1146	<0.001
Age 69	2.94 (1.85, 4.78), *n* = 1028	2.72 (1.69, 4.15), *n* = 1076	<0.005
Word memory, mean ± SD, n			b
Age 43	24.1 ± 6.1, *n* = 1335	25.7 ± 6.4, *n* = 1385	<0.001
Age 53	23.0 ± 6.2, *n* = 1397	24.9 ± 6.2, *n* = 1473	<0.001
Age 60–64	23.0 ± 5.9, *n* = 1023	25.4 ± 6.1, *n* = 1127	<0.001
Age 69	21.2 ± 6.0, *n* = 1005	23.1 ± 6.0, *n* = 1056	<0.001
Anthropometry^d^, mean ± SD, n			b
Height (m)	1.75 ± 0.06, *n* = 1409	1.62 ± 0.06, *n* = 1485	<0.001
BMI (kg/m^2^)	27.4 ± 4.0, *n* = 1408	27.4 ± 5.4, n = 1473	0.83
Behavioural risk factors^d^, n (%)			
Leisure time physical activity			c
None	665 (47.0)	754 (50.4)	0.09
1–4 times/month	267 (18.9)	242 (16.2)	
5+ times/month	484 (34.2)	500 (33.4)	
Smoking status			c
Current	329 (23.2)	338 (22.6)	<0.001
Previous smoker	728 (51.3)	669 (44.7)	
Never smoker	361 (25.5)	489 (32.7)	
Health status^d^, n(%) or mean ± SD			c
History of diabetes	45 (3.0)	39 (2.5)	0.44
History of CVD events	83 (5.9)	46 (3.2)	<0.001
Experiencing respiratory symptoms	276 (19.5)	274 (18.3)	0.42
Experiencing knee pain	217 (15.5)	306 (20.7)	<0.001
Symptoms of depression/anxiety	15.6 ± 8.5, *n* = 1386	18.9 ± 10.3, *n* = 1463	<0.001^b^
Highest household occupational class^d^, n(%)			c
I Professional/II intermediate	781 (52.1)	559 (36.4)	<0.001
III Skilled (non-manual or manual)	566 (37.7)	655 (42.6)	
IV Partly skilled/V unskilled	153 (10.2)	323 (21.0)	
Educational attainment^e^, n(%)			c
Degree or higher	211 (14.7)	81 (5.5)	<0.001
GCE A level or Burnham B	405 (28.2)	343 (23.5)	
GCE O level or Burnham C	207 (14.4)	377 (25.6)	
Sub GCE	91 (6.3)	133 (9.1)	
None attempted	521 (36.3)	528 (36.1)	

a Kruskal-Wallis equality of populations rank test for non-parametric data.

b One-way ANOVA.

c Chi-square test.

d At age 53 years.

e At age 26 years.

### 3.2 Autoregressive cross-lagged model

**Fig. 2:**
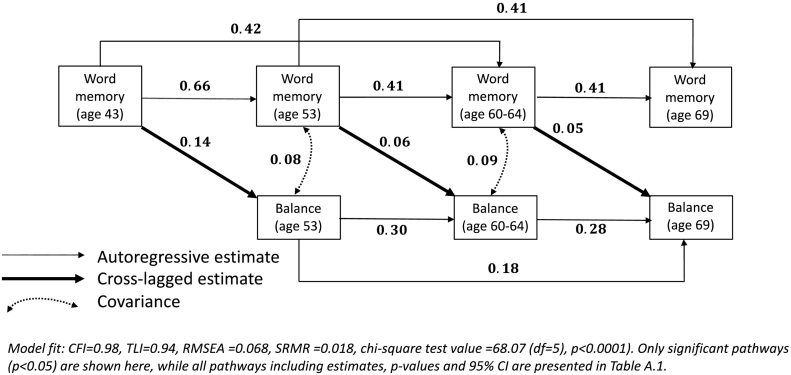
Autoregressive cross-lagged model of the associations between word memory and balance.

A total of 2735 individuals had at least one lagged association between memory and balance at any age and in either direction and thus were included in the autoregressive cross-lagged model. Satorra-Bentler scaled chi-square difference tests revealed no sex differences in pathways, thus the main model results include males and females. All presented estimates are standardised. Autoregressive effects suggested low-to-moderate stability of balance over time [β = 0.30 (95%CI: 0.24,0.36), 0.28(0.20,0.36)] and moderate-to-high stability of word memory [β = 0.66(0.63,0.68), 0.41(0.37,0.45), 0.41(0.37,0.45)] (Fig. 2, Table A.1)]. There was evidence of a cross-lagged association between word memory and subsequent balance at all ages, but no association of balance performance with subsequent word memory at any age.

The effect size was bigger for word memory at age 43 to balance time at age 53 [β = 0.14(0.10,0.17); *p* < 0.001] than for associations with balance time at ages 60–64 [β = 0.055(0.012,0.098)] or 69 [β = 0.05(0.01,0.09)]. There was no difference in effect size between the two later ages. Covariance between balance and word memory suggested a correlation at ages 53 and 60–64 (*p* < 0.001), however, this weakened over time and was no longer present at age 69 (*p* = 0.7). The model was an excellent fit (CFI = 0.98, TLI = 0.94, RMSEA = 0.068, SRMR = 0.018) (see Fig. 2).

### 3.3 Dual change score models

Due to the complexity of the dual change score models, they failed to converge in the main sample, forcing the sample to be restricted to those with complete data on balance time and word memory at all ages (*n* = 1641). As expected, the freely estimated model (Model 1) had the best fit. Notably, the unidirectional word memory to balance model (Model 3) also demonstrated an excellent fit for the data (Table 2) and across all six model fit indices, had a similar fit to the freely estimated model. Conversely, the no-coupling model (Model 2) and the unidirectional balance to word memory model (Model 4) were similar to one another and were a worse fit compared to Models 1 and 3 (Table 2). The optimal fit of Model 3 and the poor fit of Models 2 and 4 indicate support for a unidirectional relationship between word memory and subsequent change in balance.

**Table 2:** Model fit parameters for dual change score model between balance ability and word memory from age 53 to 69, complete cases only (*n* = 1641).

Model fit parameter	Adequate fit	Excellent fit	Model 1: Freely estimated	Model 2: No coupling	Model 3: VM➔ Bal only	Model 4: Bal➔ VM only
CFI	≥0.90	≥0.95	0.99	0.96	0.99	0.96
TLI	≥0.90	≥0.95	0.97	0.93	0.97	0.91
RMSEA	≤0.08	≤0.06	0.046	0.069	0.041	0.077
SRMR	≤0.08	≤0.06	0.032	0.084	0.032	0.084
AIC	The model with a lower AIC/ BIC of ≥10 is more likely to be the true model (e.g. a better fit)		−3727.874	−3728	−3680	−3728
BIC			−3609.007	−3609	−3586	−3620

CFI = Comparative Fit Index; TLI = Tucker-Lewis Index; RMSEA = Root Mean Square Error of Approximation; SMR = Standardised Root Mean Square Residual; AIC = Akaike Information Criterion; BIC=Bayesian Information Criterion.

### 3.4 Random-effects models

As both the autoregressive cross-lagged model and the dual change score model suggested a unidirectional association between word memory and subsequent balance performance, word memory (at ages 43, 53 and 60–64) was considered as the independent variable and balance performance (at ages 53, 60–64 and 69) was the dependent variable in lagged random-effects models. Individuals were included if they had at least one measure of word memory at age *t* and balance at age *t* *+* *1* (*n* = 2783). Due to the skewed balance times, balance was log-transformed. In a sex-adjusted model, a 1SD increase in word memory was associated with a 9% (95% CI: 7, 12%) increase in subsequent balance time (Table 3). The negative age-interaction suggested that this association became smaller with age (e.g. by −0.4% (−0.6, −0.1) per year).

**Table 3:** Associations between word memory (per 1SD) and subsequent log-transformed balance time (s) in random-effects models.

Model:	Percent change in lagged ln (balance time) at age 53 per SD of word memory [intercept]		Word memory (SD) *age (year) interaction [slope]	
	Coefficient (%) (95% CI)	*p*-Value	Coefficient (%) (95% CI)	*p*-Value
Maximal available sample n = 2783 (obs = 6195)				
0: age^a^, sex^b^	9 (7, 12)	<0.001	−0.4 (−0.6, −0.1)	<0.005
Complete cases sample *n* = 2551 (obs = 5167)				
1: age, sex^b^	9 (7, 12)	<0.001	−0.4 (−0.7, −0.2)	<0.005
2: model 1 + death + attrition^c^	9 (6, 11)	<0.001	−0.4 (−0.6, −0.1)	<0.005
3: model 2 + anthropometric^d^	8 (6, 11)	<0.001	−0.4 (−0.6, −0.2)	<0.005
4: model 3 + health status^e^	8 (6, 11)	<0.001	−0.4 (−0.6, −0.2)	<0.005
5: model 3 + health behaviours^f^	7 (5, 10)	<0.001	−0.4 (−0.6, −0.2)	<0.005
6: model 3 + SEP^g^	7 (4, 9)	<0.001	−0.4 (−0.6, −0.2)	<0.005
7: model 3 + education^h^	4 (1, 7)	<0.01	−0.2 (−0.5, 0.1)	0.13
8: fully adjusted^i^	3 (0.1, 6)	0.04	−0.2 (−0.5, 0.1)	0.13

a Age is centred at age 53 = 0 in all models.

b Adjusted for age, sex, age*sex.

c Adjusted for model 1 + death, attrition.

d Adjusted for model 2 + height, height^2^, BMI.

e Adjusted for model 3 + respiratory symptoms, knee pain, history of diabetes, history of cardiovascular events, symptoms of anxiety/depression, age*knee pain, age*symptoms of anxiety/depression.

f Adjusted for model 3 + smoking history, leisure time physical activity, age* leisure time physical activity.

g Adjusted for model 3 + maternal education, paternal social class, adulthood social class, age*maternal education, age*paternal social class.

h Adjusted for model 3 + education, age*education.

i Adjusted for all covariates in Models 1–7.

Adjustment for death, attrition, anthropometric, health and behavioural factors had little impact on the estimates (Table 3). Education partly explained the association between word memory and balance as indicated by partial attenuation of both the intercept and slope estimates. In the fully-adjusted model, a 1SD change in word memory remained associated with a 3% (0.1,6%) change in balance time.

## 4 Discussion

In a large nationally representative study with multiple measurements of word memory and balance performance over 26 years, there was consistent evidence that higher word memory was associated with better subsequent balance performance. This association was strongest in midlife and weakened with age, with no evidence of associations operating in the opposite direction. When the model was adjusted for covariates, only education had a meaningful impact on estimates. These results suggest that cognition, as assessed by word memory, may play an important role in balance ability, with educational attainment partially explaining this association.

In previous longitudinal studies, balance performance is most commonly modelled as a function of cognitive ability (Kuh et al., 2009; Kuh et al., 2006; Saverino et al., 2016; Tabbarah et al., 2002), with few studies examining balance and subsequent cognitive outcomes (Bullain et al., 2016; Ursin et al., 2015) and only three studies investigating bidirectional balance-cognition associations (Chen et al., 2016; Finkel et al., 2016; Tolea et al., 2015). One study visually compared estimates from two separate unidirectional regression models concluding that baseline balance ‘predicted’ cognitive function at a later age (Chen T 2016), while another study concluded that baseline cognitive impairment had a unidirectional association with declining balance-gait performance (Tolea et al., 2015); as such, neither study appropriately addressed the question of directionality. As above, the third study applied bivariate dual change score models to capture the extent to which change in balance or cognition was a function of the other and identified an association between balance performance and subsequent changes in processing speed (Finkel et al., 2016). These studies examined older samples (mean age 65 or 75 at baseline) (Chen et al., 2016; Finkel et al., 2016). Age differences may explain the contrasting findings, especially as our study findings suggest that the association may be age-dependent. The studies were conducted in the USA, Sweden and Japan and included adults born earlier in the 20th century than the NSHD cohort. Thus, differences may also be a result of country or cohort effects, especially as there are regional and temporal differences in longevity and patterns of age-related change in cognitive and physical capability (Ahrenfeldt et al., 2018; Varnum et al., 2010).

Previous work from our group has suggested that childhood cognitive ability is associated with midlife balance performance (Blodgett et al., 2020b), although this association was primarily explained by education, social class and adult cognition. Here, we extend these results to suggest that adult cognitive ability plays an important role in balance performance, with education only partially explaining the association.

Cognitive integration of sensory input and motor output is crucial for postural equilibrium (Menant et al., 2014). Individuals with higher cognitive ability, in particular working memory, often have greater white matter volume (Lazar, 2017), increased hippocampal activity (Leszczynski, 2011) and sustained activation in the prefrontal cortex (Funahashi, 2017); this neural activity is relevant to maintaining balance. Current evidence has not distinguished if memory plays a direct role in balance performance or if both abilities rely on functionality of shared neural structures. Our findings suggest that components of memory may be involved in balance mechanisms, rather than an age-related correlation between the neural structure and functions involved in both processes.

In addition to the physiological mechanism of association between word memory and balance ability, education may play a role. Education can beneficially impact cognitive ability; equally, higher cognitive ability can lead to higher educational attainment (Ritchie and Tucker-Drob, 2018). Higher educational attainment is linked to better health status, which may be a result of better access to healthcare or improved health behaviours and has a positive association with balance performance (Blodgett et al., 2020a; Hahn and Truman, 2015).

Consistent across models, the association between word memory and balance was strongest in midlife and weakened with age. While cognitive processing involved in balance remains important in later life, physical health-related factors may become more dominant. For example, as musculoskeletal problems (e.g. hip/knee pain, sarcopenia) become more common with age (Anderson and Anderson and Loeser, 2010; Walston, 2012), changes in physical health at older ages may have a greater impact on balance than cognitive pathways. Previous evidence in NSHD is consistent with this explanation, demonstrating that associations between knee pain and balance performance increase with age (Blodgett et al., 2020a). As balance ability relies on input from visual, vestibular and proprioceptive sources, age-related changes in the ability to perceive these sensory inputs could also explain a weakening association between cognition and balance at older ages. Given that balance may have a greater reliance on visual input at older ages (Púcik et al., 2014; Saftari and Kwon, 2018; Yeh et al., 2014), one would expect that visual acuity would explain more variation in balance performance compared with cognitive processes. Replication of these analyses in older adults with eyes open tests may inform how the roles of visual input and subsequent processing in balance change with age.

This is one of few studies to specifically examine the direction of associations between word memory and balance. It utilises more than 25 years of data on word memory and 15 years of balance data in 3000 individuals. Using repeated measures of word memory and balance performance and approaches which model bidirectional associations simultaneously, we have overcome limitations in analytical methodology of previous studies. This is the first study to examine the directionality in associations between cognitive and balance abilities in an age homogenous sample in mid-life, thus identifying changes with age without any confounding by age or birth cohort. There are limitations to our analyses. Despite the advantage of repeated measures of balance, with only three time points, balance performance could only be modelled linearly in the dual change score and random-effects models. Utilising data with more waves of follow-up could allow piecewise trajectories that reflect potential changes in cognitive-balance mechanisms with age to be considered. The autoregressive cross-lagged models enabled four measures of cognition to be included, while equal numbers of each measure were required for the dual change score models. Visual acuity at relevant ages in adulthood has not been assessed in the main NSHD sample, thus we were unable to investigate how vision may have contributed to these associations and their changes with age.

Another important limitation is missing data both from individuals lost to follow-up before age 43 and incomplete data between ages 43 and 69; this is important in interpretation of dual change score models whereby only individuals with complete data on word memory and balance tests could be included. Across all models, the sample size was maximised, resulting in slightly different samples for each model, however findings were consistent across all models despite different sample sizes. Importantly, for all comparisons between pathways (auto-regressive cross-lagged model), models (dual change score model) and stages of adjustment (random-effects model), the same sample was compared. Representativeness of the NSHD cohort has been extensively examined (Kuh et al., 2016; Stafford et al., 2013), demonstrating that those lost to follow-up are more likely to have poor physical and cognitive health compared to those who remained in the study. Individuals with complete cognitive and balance data also tend to perform better than those with any missing data. Thus, it is hypothesised that the associations assessed here are conservative and potentially underestimated the true strength of association. Finally, word memory may have been measured with more precision than balance time due to differences in test sensitivity; replication of analyses with a more sensitive balance test (e.g. postural sway as assessed by a force plate) is recommended.

## 5 Conclusions

This paper provides evidence of a unidirectional association between word memory and balance and suggests that education may partly explain this association. Evidence in this research area remains limited; further investigation into these relationships are required and replication of findings is critical. Future research should examine longer trajectories from early to late adulthood and across a wider range of cognitive measures to assess if these associations differ with age (as suggested here) or by cognitive domain. Many cohort studies have collected data on balance and cognitive performance across numerous waves; secondary analysis of these data would be valuable in contributing to the current evidence on the topic.

The association between word memory and subsequent balance performance may have later implications for interventions aimed at improving balance ability. Interventions tend to be limited to physical training programs such as flexibility, balance or resistance training. Few interventions have included a cognitive component, although there is evidence suggesting that dual or multi-task training (that combines cognitive and physical training) can improve balance ability and minimise falls risk (Halvarsson et al., 2015; Halvarsson et al., 2013). Word memory may be a proxy for overall cognitive function; further research must assess if findings can be extended to other cognitive areas to better understand which cognitive processes contribute to changes in balance with age. This, along with insights from functional magnetic resonance imaging data during one-legged balance tests, could aid in the design of cognitive training that targets balance ability.

## Funding

This work was supported by the 10.13039/501100000024Canadian Institutes of Health Research (FDSA), the Canadian Centennial Scholarship Fund, the 10.13039/501100000265Medical Research Council (MC_UU_00019/1 Theme 1: Cohorts and Data Collection to NSHD; MC_UU_12019/1, MC_UU_12019/2 and MC_UU_12019/4), the 10.13039/501100000269Economic and Social Research Council (ES/K000357/1) and the 10.13039/100010269Wellcome Trust (WT107467).

## Data available

The datasets used in this study will not be made publicly available. Access to NSHD data adheres to strict confidentiality guidelines but these data are available to bona fide researchers upon request to the NSHD Data Sharing Committee via a standard application procedure. Further details can be found at http://www.nshd.mrc.ac.uk/data. doi: https://doi.org/10.5522/NSHD/Q101; doi: https://doi.org/10.5522/NSHD/Q102; doi: https://doi.org/10.5522/NSHD/Q103.

## CRediT authorship contribution statement

**Joanna M Blodgett:** Conceptualization, Methodology, Formal analysis, Visualization, Writing - Original Draft, Writing - Review & Editing. **Rachel Cooper:** Conceptualization, Writing - Review & Editing, Supervision. **Daniel HJ Davis:** Conceptualization, Writing - Review & Editing, Supervision. **Diana Kuh:** Conceptualization, Writing - Review & Editing, Supervision. **Rebecca Hardy:** Conceptualization, Methodology, Writing - Review & Editing, Supervision.

## Declaration of competing interest

None.

## Figures and Tables

**Table A.1:** Standardised results from the autoregressive cross-lagged model of balance and word memory, n = 2735; corresponding estimates in Fig. 2.

Dependent variable	Independent variable	Change in standardised balance per 1SD change in cognition (95% CI)	*p*-Value
Autoregressive estimates			
Balance at age 60–64	*ON* Word age 53	0.30 (0.24, 0.36)	<0.001
Balance at age 69	*ON* Word age 60–64	0.28 (0.20, 0.36)	<0.001
	*ON* Word age 53		<0.001
Word memory at age 53	*ON* Word memory age 43	0.66 (0.63, 0.68)	<0.001
Word memory at age 60–64	*ON* Word memory age 53	0.41 (0.37, 0.45)	<0.001
	*ON* Word memory age 43	0.42 (0.38, 0.46)	<0.001
Word memory at age 69	*ON* Word memory age 60–64	0.41 (0.37, 0.45)	<0.001
	*ON* Word memory age 53	0.41 (0.36, 0.45)	<0.001
Cross-lagged estimates			
Balance at age 53	*ON* Word memory age 43	0.14 (0.10, 0.17)	<0.001
Balance at age 60–64	*ON* Word memory age 53	0.06 (0.01, 0.10)	0.01
Balance at age 69	*ON* Word memory age 60–64	0.05 (0.01, 0.09)	0.02
Word memory at age 60–64	*ON* Balance age 53	0.01 (−0.02, 0.04)	0.49
Word memory at age 69	*ON* Balance age 60–64	0.01 (−0.03, 0.04)	0.77
Covariance estimates			
Word memory age 53	*WITH* Balance age 53	0.08 (0.04, 0.13)	<0.001
Word memory age 60–64	*WITH* Balance age 60–64	0.09 (0.04, 0.13)	<0.001
Word memory age 69	*WITH* Balance age 69	0.01 (−0.04, 0.05)	0.69
